# Parameter optimization of logistic regression classifiers

**DOI:** 10.1186/1471-2202-14-S1-P62

**Published:** 2013-07-08

**Authors:** Jason S Sherwin, Josh Chartier

**Affiliations:** 1 Department of Biomedical Engineering, Columbia University, New York, NY 10025, USA; 2 Human Research and Engineering Directorate, U.S. Army Aberdeen Proving Ground, Aberdeen, MD 21005, USA; 3 Department of Biomedical Engineering, Rice University, Houston, TX 77005, USA

## 

Logistic regression (LR) classifiers have been used successfully in the single-trial analysis of EEG data, especially in tasks of perceptual decision-making [1,2], but heuristics govern the choices for classifier parameters, such as window size (δ). Furthermore, no rigorous definition exists as to the number of epochs (N) of either class that would allow sufficient classifier training before testing using leave-one-out cross-validation. Here, we attempt to address these issues by exploring this discrete parameter space with the aid of a genetic algorithm. In doing so, we draw preliminary conclusions on both subject-specific and subject-general trends of these classifiers.

To establish a baseline for comparison, we utilize EEG data from a previous study using LR to classify neural response to a two-choice forced-decision face vs. car visual task [1]. In this study, a window size (δ) of 60 ms was used to segment epochs for classification. Other studies using this technique also employ a comparable window size [2,3], even though δ has the potential to drastically affect classifier training and performance. Similarly, the number of epochs used to train the classifier can greatly affect its performance, a number too low causing an insufficient number of points through which a dividing hyperplane can be found.

Recognizing the dependence of classifier performance on these discrete parameters, we use a genetic algorithm to explore the δ vs. N design space. In doing so, we track an objective function whose value depends on maximizing an epoch window's leave-one-out A_z _(area under receiver-operating characteristic) value while decreasing its variability (determined from bootstrapping), which increases with a low number of epochs. Once converging to subject-specific values of δ* and N*, we then test the classifier solution for statistical significance using the false discovery rate across all windows [4], as there are approximately E/2δ* multiple comparisons for an E milliseconds epoch with 50% window overlap.

First, minimizing our objective function with N held constant at its maximum, we find that δ* can be tuned in a subject-specific way and we find on average a 3.7 ± 1.1% improvement in maximum A_z _from that of the earlier study. Second, we vary δ (δ ∈ [5, 6, ..., 149, 150]ms) and N (N ∈ [10, 11, ..., N_max_-1, N_max_] ) simultaneously and converge using a genetic algorithm (6-bit resolution, 36-member population, 0.7 crossover probability, 0.7/(population size) mutation probability, [5]) to a subject-specific δ* and N*. In each subject but one we find that N* < N_max _and that δ* is a subject-specific parameter that differs from the heuristics offered by previous work. Finally, on a group level, we find that the components of our objective function exhibit distinct variation with respect to δ and N, with an epoch's maximum A_z _optimizing for low N and low δ, while its A_z _variability minimizes for high N and maximizes for low N, nearly irrespective of δ.
